# Polypharmacy, benzodiazepines, and antidepressants, but not antipsychotics, are associated with increased falls risk in UK care home residents: a prospective multi-centre study

**DOI:** 10.1007/s41999-020-00376-1

**Published:** 2020-08-19

**Authors:** Madeline A. D. Izza, Eleanor Lunt, Adam L. Gordon, John R. F. Gladman, Sarah Armstrong, Pip Logan

**Affiliations:** 1 School of Medicine, University of Nottingham, Nottingham, UK; 2 Nottingham NIHR Biomedical Research Centre – Musculoskeletal Theme, Nottingham, UK; 3 NIHR Applied Research Collaboration East Midlands (ARC-EM), Nottingham, UK; 4 Nottingham CityCare Partnership, Nottingham, UK

**Keywords:** Residential facilities, Accidental falls, Polypharmacy, Psychotropic medications

## Abstract

**Purpose:**

Falls and polypharmacy are both common in care home residents. Deprescribing of medications in residents with increased falls risk is encouraged. Psychotropic medications are known to increase falls risk in older adults. These drugs are often used in care home residents for depression, anxiety, and behavioural and psychological symptoms of dementia. However, a few studies have explored the link between polypharmacy, psychotropic medications, and falls risk in care home residents.

**Methods:**

This was a prospective cohort study of residents from 84 UK care homes. Data were collected from residents’ care records and medication administration records. Age, diagnoses, gender, number of medications, and number of psychotropic medications were collected at baseline and residents were monitored over three months for occurrence of falls. Logistic regression models were used to assess the effect of multiple medications and psychotropic medication on falls whilst adjusting for confounders.

**Results:**

Of the 1655 participants, mean age 85 (SD 8.9) years, 67.9% female, 519 (31%) fell in 3 months. Both the total number of regular drugs prescribed and taking ≥ 1 regular psychotropic medication were independent risk factors for falling (adjusted odds ratio (OR) 1.06 (95% CI 1.03–1.09, *p* < 0.01) and 1.39 (95% CI 1.10–1.76, *p* < 0.01), respectively). The risk of falls was higher in those taking antidepressants (*p* < 0.01) and benzodiazepines (*p* < 0.01) but not antipsychotics (*p* > 0.05).

**Conclusion:**

In UK care homes, number of medications and psychotropic medications (particularly antidepressants and benzodiazepines) predicted falls. This information can be used to inform prescribing and deprescribing decisions.

## Key summary points

**Aim:**

To explore the link between polypharmacy, psychotropic medications, and falls risk in a cohort of UK care home residents.

**Findings:**

Polypharmacy and psychotropic drugs are predictive of falls in UK care home residents.

**Message:**

Deprescribing interventions relating to psychotropic drugs should continue to be encouraged.

## Introduction

Falls are associated with polypharmacy and psychotropic drug use. Taking four or more medications is associated with increased fall rates by 18% in those over the age of 60 [1]. Older people living at home who take psychotropic drugs are up to 1.62 times more likely to fall [2]. These associations are often used to justify routine medication reviews and deprescribing in older people at risk of falls [3].

Falls incidence in older people living in care homes is three times that of those living at home. Contributors include physical frailty, cognitive impairment, comorbidities, and polypharmacy, which are all more prevalent in care homes [4]. Psychotropic medications (including, antipsychotics, anxiolytics, hypnotics, and antidepressants) are more commonly prescribed for care home residents because of underlying dementia, depression, and behavioural symptoms [5, 6]. The average UK care home resident takes eight medications, which could place them at increased risk of falls, although association between falls and medications in this group has been less widely studied than community populations [2, 7].

Polypharmacy and psychotropic drug use might be expected to show a similar association with falls in care home and community-dwelling populations. However, the risk factors for falls in care homes may differ from the wider population, and the association between falls, polypharmacy, and psychotropic drug use may differ accordingly. Doctors may be tempted to prescribe psychotropic medications to reduce behaviours such as wandering, which are felt to be risk factors for falls, or they may be unsure about the size of risk of falls associated with them. Psychotropic drug prescriptions in Dutch Nursing Home residents are associated with a threefold increase in falls risk [8]. Dutch Nursing Homes are, though, organised differently from UK care homes and, importantly, have differing practises regarding physical restraint—so associations seen in one country may not be replicated elsewhere [9].

To provide clarity on this topic and support better clinical decision-making, we examined the relationship between falls, polypharmacy and psychotropic drug use, using data from a large cluster randomised controlled trial of a multifactorial falls prevention programme in care homes (FinCH). The study objectives were to determine if polypharmacy and psychotropic medications increased the risk of falls in UK care home residents.

## Methods

The Falls in Care Home (FinCH) study was a multi-centre cluster randomised controlled trial investigating the efficacy of the Guide to Action Care Home (GtACH) fall prevention programme. It was conducted between 2016 and 2019 in 84 UK care homes. The full protocol has been published [10]. Ethical approval was provided by the Yorkshire & The Humber-Bradford Leeds Research Ethics Committee (REC: 16/YH/0111). Permission to use data for the sub-analysis presented here was granted by the Trial Steering committee, shown in “Appendix A”. Informed consent for the trial was obtained from participants, or a consultee if participants lacked capacity.

This sub-analysis considers the baseline 3 month period prior to randomisation and hence before exposure to any study-related intervention which might have changed falls or medication management. Data collection took place in the 18 months between August 2016 and February 2018.

Data were collected from care home records on age, gender, and medical diagnoses. Medications were recorded from care home Medicine Administration Record (MAR) charts. Eye drops and nasal sprays were included, because they are often systemically absorbed and may contribute to drug interactions. Over-the-counter (OTC) medications were included. Pro re nata (PRN) medications (taken as needed) were analysed separately for their contribution to medication burden. Medical equipment and dressings, topical medications (except eye drops and nasal sprays), vaccinations, and dietary supplements were excluded. Medication inclusion or exclusion decisions were considered independently by two researchers to minimise error. The Anatomical Therapeutic Chemical (ATC) classification system was used to classify psychotropic drugs and create an adapted version of the classification used by Cox et al. [8]. This classification and the psychotropic drugs prescribed are shown in “Appendix B”. Polypharmacy was defined as taking five or more medications based on prior work which shows a strong association with falls at this level [11]. Falls incidence over the 3 month observation period was the outcome variable. Falls were defined as “an unexpected event in which the participants come to rest on the ground, floor, or lower level” [12]. Due to uncertainty associated with unwitnessed falls, if a resident was found on the floor, it was assumed that they had fallen. Falls data were collated from care home records and incident report forms, both of which have been previously validated as reliable sources [13].

Demographic, medication, and falls data for each participant in the baseline period were imported into Microsoft Excel 2016. Falls without a date were excluded. Where medication data were affected by spelling errors, they were included only if an agreement was established between two medically trained members of the research team over which drug was intended.

Descriptive statistics were calculated for the total population, fallers and non-fallers. Categorical data were expressed as frequencies and percentages. Histograms were used to determine distribution and continuous data were expressed as means with standard deviations (SDs) if normally distributed, or medians with interquartile ranges (IQRs) if non-parametric. Any data identified as extreme outliers were removed. Odds ratios and mean differences were calculated to determine the significant characteristics between fallers and non-fallers.

A logistic regression model with the dichotomous dependent variable of faller or non-faller and the continuous predictor variable of number of drugs (as a measure of polypharmacy) was fitted. Demographic data were included as predictors in the model to control for possible confounders. A further binary logistic regression was performed using the same dichotomous dependent variable and adding in whether the participant was on psychotropic drug(s) or not as a categorical predictor variable. Each regression analysis was performed twice, with and without PRN drugs. A *p* value < 0.05 was considered significant. All statistical analyses were carried out using Statistical Package for Social Sciences (SPSS, version 24.0, IBM Corporation, Armonk, NY).

## Results

### Study population

Data from 1655 participants were analysed, with characteristics summarised in Table 1. The mean age of participants was 85 years (SD 8.9). Approximately two-thirds of the population were female. Dementia was the most prevalent diagnosis with 1112 residents (67%) diagnosed with the condition.

**Table 1:** Characteristics of study population, showing the total population and the differences between fallers and non-fallers

	Total population	Fallers	Non-fallers	Univariate analysis	*P* value
	*N* = 1655	*N* = 519	*N* = 1136	OR (95% CI)	
Age	85.0 (SD 8.9)	85.8 (SD 8.1)	84.7 (SD 9.2)	1.10 (0.18–2.02)^a^	0.019
Gender					
Female	1,123 (67.9%)	329 (63.4%)	794 (69.9%)		
Male	532 (32.1%)	190 (36.6%)	342 (30.1%)	1.34 (1.08–1.67)	0.008
Diagnoses					
Dementia	1112 (67.2%)	386 (74.4%)	726 (63.9%)	1.64 (1.30–2.06)	< 0.001
Diabetes	321 (19.4%)	98 (18.9%)	223 (19.6%)	0.95 (0.73–1.24)	0.721
Stroke	262 (15.8%)	81 (15.6%)	181 (15.9%)	0.97 (0.73–1.30)	0.866
Coronary heart disease	235 (14.2%)^b^	73 (14.1%)	162 (14.3%)^b^	0.98 (0.73–1.32)	0.910
Number of drugs					
Without PRN	6 (IQR 3–9)	6 (IQR 4–9)	6 (IQR 3–8)		<0.001
Including PRN	7 (IQR 4–10)	8 (IQR 5–11)	7 (IQR 4–10)		<0.001
Number of residents on polypharmacy^c^					
Without PRN	1,024 (61.9%)	350 (67.4%)	674 (59.3%)	1.42 (1.14–1.77)	0.002
Including PRN	1,164 (70.3%)	398 (76.7%)	766 (67.4%)	1.59 (1.25–2.02)	< 0.001
Number of residents taking psychotropic drug(s)					
Without PRN	816 (49.3%)	295 (56.8%)	521 (45.9%)	1.55 (1.26–1.92)	< 0.001
Including PRN	870 (52.6%)	307 (59.2%)	563 (49.6%)	1.47 (1.19–1.82)	< 0.001
Number of residents taking antidepressant(s)					
Without PRN	613 (37%)	219 (42.2%)	394 (34.7%)	1.37 (1.11–1.70)	0.003
Including PRN	614 (37.1%)	219 (42.2%)	395 (34.8%)	1.37 (1.12–1.69)	0.004
Number of residents taking antipsychotic(s)					
Without PRN	251 (15.2%)	87 (16.8%)	164 (14.4%)	1.19 (0.90–1.59)	0.221
Including PRN	278 (16.8%)	91 (17.5%)	187 (16.5%)	1.08 (0.82–1.42)	0.588
Number of residents taking benzodiazepine(s)					
Without PRN	207 (12.5%)	84 (16.2%)	123 (10.8%)	1.59 (1.18–2.14)	0.002
Including PRN	329 (19.9%)	122 (23.5%)	207 (18.2%)	1.38 (1.07–1.77)	0.013

Values are Mean (SD), Median (IQR) or Number (Proportion)

^a^Mean difference calculated for continuous data

^b^In calculating coronary heart disease percentages *n* = 1654 (Total population) and *n* = 1135 (Non-Fallers) to account for missing data for one resident who did not fall

^c^Polypharmacy = ≥ 5 drugs

A total of 3671 (26%) medications listed in the MAR were removed due to exclusion criteria and 14 due to indecipherable spelling errors, leaving 10,226 medications used by the population which were then analysed. The median number of drugs prescribed per resident was 6 (IQR 3–9) excluding PRN. Polypharmacy (without PRN) was present in 1024 (62%) participants. Regular psychotropic drug prescriptions were present in approximately half of the population (49%).

### Polypharmacy and falls

There were a total of 1188 falls in the population, with 519 residents having ≥ 1 fall, in the 3 months. The first logistic regression model assessed the effect of the number of drugs (excluding PRN drugs), adjusting for gender, age, and dementia (Table 2). Further control for other demographics had no significant effect and was removed from the model. The model explained between 3.1% and 4.3% of variability in falls outcomes.

**Table 2:** Logistic regression analysis assessing the predictive ability of number of drugs excluding PRN drugs on fall outcomes in care home residents

Predictor	Odds ratio	95% CI for odds ratio		*P*
		Lower	Upper	
Constant	0.033			< 0.001
Age	1.021	1.008	1.034	0.001
Gender	1.405	1.120	1.762	0.003
Dementia	1.749	1.382	2.213	< 0.001
No. drugs	1.058	1.031	1.086	< 0.001

*Note* Cox & Snell *R*^2^ = 0.031, Nagelkerke *R*^2^ = 0.043. Gender: 1 = male, 0 = female

All four predictor variables made a significant contribution to the model. For every additional drug prescribed, the odds of falling increased by 1.058 times, after controlling for the other factors mentioned above. Older residents were more likely to fall than their younger counterparts. Men were predicted to fall more than women, and residents with dementia were predicted to fall 75% more than those without dementia. In the second binary logistic regression model the predictor effect including PRN drugs was tested, this found similar results to the previous model.

### Psychotropic medications and falls

816 (49.3%) residents took regular psychotropic medications. In unadjusted analysis, psychotropic medications were associated with falls (*p* < 0.001). The risk of falls was higher in those taking antidepressants (*p* < 0.01) and benzodiazepines (*p* < 0.01) but not antipsychotics (*p* > 0.05).

A third binary logistic regression model assessed the effect of one or more psychotropic drugs excluding PRN drugs, and controlled for age, gender, dementia diagnosis, and number of drugs (Table 3). The adjusted odds ratio of taking one or more psychotropic drug on falls risk was 1.39 (95% CI 1.100–1.762, *p* = 0.006). All variables controlled for showed a similar significant effect as in previous models. Likewise, the number of drugs remained a significant predictor.

**Table 3:** Binary logistic regression analysis assessing the predictive ability of psychotropic drug(s) excluding those indicated as PRN, on fall outcomes in care home residents

Predictor	Odds ratio	95% CI for odds ratio		*P*
		Lower	Upper	
Constant	0.027			< 0.001
Age	1.023	1.010	1.036	0.001
Gender	1.413	1.126	1.774	0.003
Dementia	1.686	1.330	2.138	< 0.001
No. drugs	1.041	1.011	1.071	0.006
Psychotropic drug(s)	1.392	1.100	1.762	0.006

*Note* Cox & Snell *R*^2^ = 0.035, Nagelkerke *R*^2^ = 0.049. Gender: 1 = male, 0 = female

In the final model, the predictor effect of whether a resident was on one or more psychotropic drugs including PRN drugs was tested, this again yielded similar results to those excluding PRN.

## Discussion

Polypharmacy and psychotropic prescriptions were both prevalent in care home residents, and both were associated with an increased falls risk. These findings were independent of age, gender, and dementia diagnosis.

An important strength of this study is the large and representative dataset of UK care home residents from the FinCH study trial. The age of residents in this study was similar to that in a previous representative cohort study on the health of UK care home residents [7]. The proportion of residents with dementia in this study is close to that reported in large national studies [7, 14]. Only one participant was excluded from this study due to missing diagnostic data. Prospective falls data were rigorously collected and recorded on standardised forms, reducing the probability of recall bias and underreporting. The use of MAR sheets, used by care home staff to administer drugs, provided reliable data on medications prescribed and taken. Limitations associated with the use of MAR sheets are that the duration of drug prescription cannot be ascertained from these and that drugs could have been changed during the three months of follow-up. It was not possible in our analysis to account fully for the influence of individual or combined comorbidities. Medical conditions, for instance arthritis, may affect postural stability and gait more than the drugs given for them. We did not include functional impairment in our model; this has the potential to confound our analysis, for example, if antipsychotics were predominantly prescribed in people who were bed or chairbound. We also did not collate data on drug dosage and this might reasonably have been expected to influence the likelihood of falls. Dementia severity was not recorded as part of the FINCH study—cognitive testing was not part of the protocol and care home records do not record dementia severity well. It is, however, unlikely that dementia severity will have confounded our analysis, as both falls and antipsychotic prescribing increase as dementia progresses, and confounding due to dementia severity would, if anything, have increased the strength of association between antipsychotic prescribing and falls.

The work replicates the findings of research undertaken in care home populations internationally that found an association between risk of falling and the number of drugs used [15, 16]. Our findings show that the association between falls and psychotropic prescriptions is as strong in the UK care home population as it is in nursing homes internationally and non-care home settings [3, 8, 17, 18]. In our study, falls were associated with antidepressants and benzodiazepines, but not with antipsychotics. Both antidepressants and benzodiazepines have been shown to increase fall risk in US nursing homes, and prescribers should include falls risk in their decision-making process when prescribing these drugs [19, 20]. Our findings should not be interpreted to conclude that antipsychotics are safe, but simply that we did not observe a risk of falls associated with them; these drugs have other risks. Although antipsychotics were prescribed to a greater proportion of fallers than non-fallers, the lack of association between falls and antipsychotics was unexpected and differed from published literature [21–23]. UK national policies, such as the National Dementia Strategy, may have led to more appropriate prescribing of antipsychotics to care home residents with multiple falls risk factors, including dementia and wandering behaviours [24].

In conclusion, our findings show that care home residents are at increased risk of falls when they take more medications and when they take antidepressants and benzodiazepines. This supports judicious deprescribing using validated tools [25, 26]. Antidepressants and benzodiazepines should be used only when absolutely indicated. They should be appropriately tapered to cessation as soon as possible. The verdict on antipsychotics is less clear, but the findings presented here are not sufficient to recommend that they are used with anything other than caution. Providing adequate training and staffing levels to support non-pharmacological approaches to both depression and behavioural symptoms in care homes seems the most rational approach. These competencies are still underdeveloped in long-term care. Building them will enable more multidisciplinary approaches to prescribing and deprescribing, ensuring that drugs are only used where they can add value, and any attendant risks are minimised [27].

## Figures and Tables

**Fig. 1:**
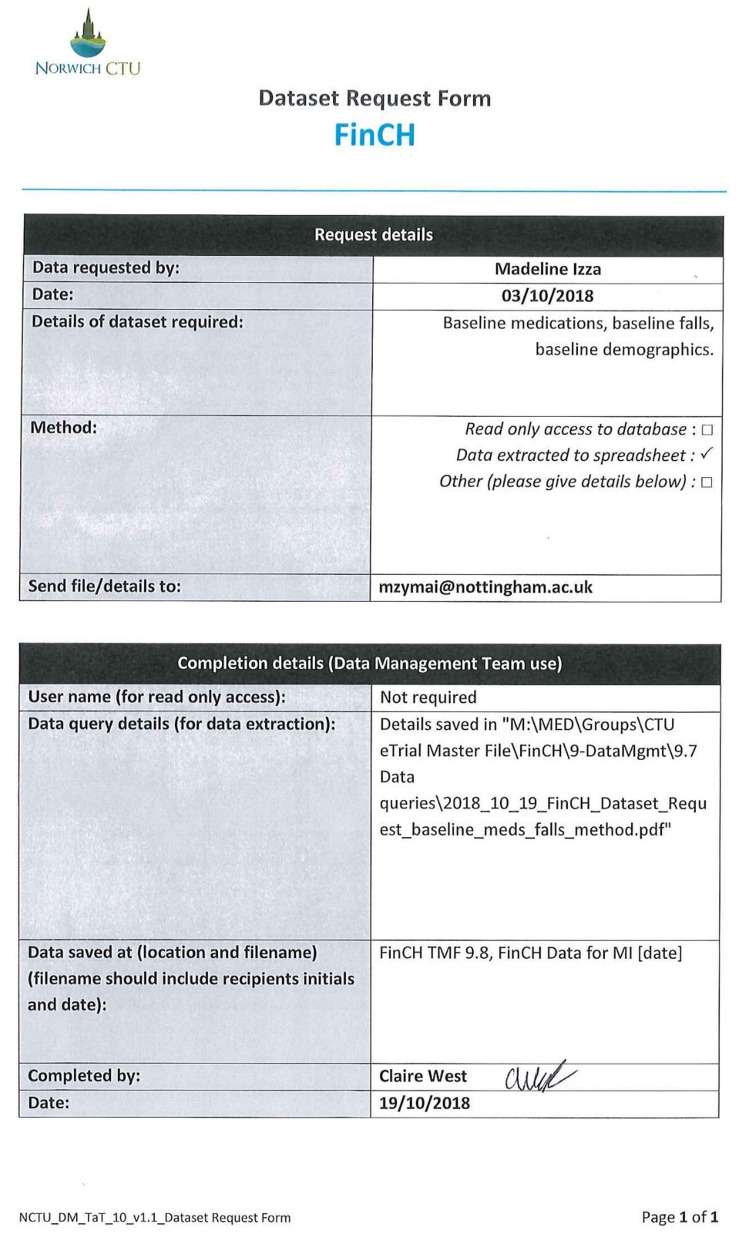
Signed data access form

## Data Availability

Data used for this study are available from Professor Logan on request.

## Appendices

### Appendix A

See Fig. 1.

### Appendix B

See Table 4.

**Table 4:** Psychotropic medication classification—those highlighted in bold were found in the data set

Name	ATC code	Class	Subgroup	Group
**Oxazepam** **Lorazepam** Lormetazepam **Temazepam** **Midazolam** Loprazolam	N05BA04 N05BA06 N05CD06 N05CD07 N05CD08 N05CD11	Short-acting benzodiazepines	Benzodiazepines	Psychotropic
**Clonazepam** **Diazepam** Chlordiazepoxide **Clobazam** Flurazepam **Nitrazepam**	N03AE01 N05BA01 N05BA02 N05BA09 N05CD01 N05CD02	Long-acting benzodiazepines		
**Zopiclone** **Zolpidem** Zaleplon	N05CF01 N05CF02 N05CF03	Benzodiazepine related drugs		
Chlorpromazine **Levomepromazine** **Promazine** Fluphenazine Perphenazine **Prochlorperazine** **Trifluoperazine** Periciazine **Haloperidol** **Benperidol** Droperidol **Flupentixol** **Zuclopenthixol** Pimozide Loxapine	N05AA01 N05AA02 N05AA03 N05AB02 N05AB03 N05AB04 N05AB06 N05AC01 N05AD01 N05AD07 N05AD08 N05AF01 N05AF05 N05AG02 N05AH01	Typical antipsychotics	Antipsychotics	
Lurasidone Clozapine **Olanzapine** **Quetiapine** Asenapine **Sulpiride** **Amisulpride** **Risperidone** **Aripiprazole** Paliperidone	N05AE05 N05AH02 N05AH03 N05AH04 N05AH05 N05AL01 N05AL05 N05AX08 N05AX12 N05AX13	Atypical antipsychotics		
**Imipramine** Clomipramine **Trimipramine** **Lofepramine** **Amitriptyline** **Nortriptyline** Doxepin **Dosulepin**	N06AA02 N06AA04 N06AA06 N06AA07 N06AA09 N06AA10 N06AA12 N06AA16	Tricyclic antidepressants	Antidepressants	
**Fluoxetine** **Citalopram** **Paroxetine** **Sertraline** Fluvoxamine **Escitalopram**	N06AB03 N06AB04 N06AB05 N06AB06 N06AB08 N06AB10	Selective serotonin reuptake inhibitors (SSRIs)		
Isocarboxazid Phenelzine Tranylcypromine	N06AF01 N06AF03 N06AF04	Monoamine oxidase inhibitors		
Moclobemide Tryptophan Mianserin **Trazodone** Minaprine Bifemelane Viloxazine **Mirtazapine** Bupropion **Venlafaxine** Reboxetine **Duloxetine** Agomelatine Vortioxetine	N06AG02 N06AX02 N06AX03 N06AX05 N06AX07 N06AX08 N06AX09 N06AX11 N06AX12 N06AX16 N06AX18 N06AX21 N06AX22 N06AX26	Other antidepressants		
